# Molecular engineering of the last-generation CNTs in smart cancer therapy by grafting PEG–PLGA–riboflavin†

**DOI:** 10.1039/d0ra07500k

**Published:** 2020-11-09

**Authors:** Somayeh Sohrabi, Mohammad Khedri, Reza Maleki, Mostafa Keshavarz Moraveji

**Affiliations:** Department of Chemical Engineering, Amirkabir University of Technology (Tehran Polytechnic) 424 Hafez Avenue Tehran 1591634311 Iran moraveji@aut.ac.ir; Computational Biology and Chemistry Group (CBCG), Universal Scientific Education and Research Network (USERN) Tehran Iran

## Abstract

In this work, the effect of environment and additives on the self-assembly and delivery of doxorubicin (DOX) have been studied. A microfluidic system with better control over molecular interactions and high surface to volume ratio has superior performance in comparison to the bulk system. Moreover, carbon nanotube (CNT) and CNT-doped structures have a high surface area to incorporate the DOX molecules into a polymer and the presence of functional groups can influence the polymer–drug interactions. In this work, the interactions of DOX with both the polymeric complex and the nanotube structure have been investigated. For quantification of the interactions, H-bonding, gyration radius, root-mean-square deviation (RMSD), Gibbs free energy, radial distribution function (RDF), energy, and Solvent Accessible Surface Area (SASA) analyses have been performed. The most stable micelle–DOX interaction is attributed to the presence of BCN in the microfluidic system according to the gyration radius and RMSD. Meanwhile, for DOX-doped CNT interaction the phosphorus-doped CNT in the microfluidic system is more stable. The highest electrostatic interaction can be seen between polymeric micelles and DOX in the presence of BCN. For nanotube–drug interaction, phosphorus-doped carbon nanotubes in the microfluidic system have the largest electrostatic interaction with the DOX. RDF results show that in the microfluidic system, nanotube–DOX affinity is larger than that of nanotube–micelle.

## Abstract 2

In this work, the effect of environment and additives on the self-assembly and delivery of doxorubicin (DOX) have been studied.

## Graphical abstract

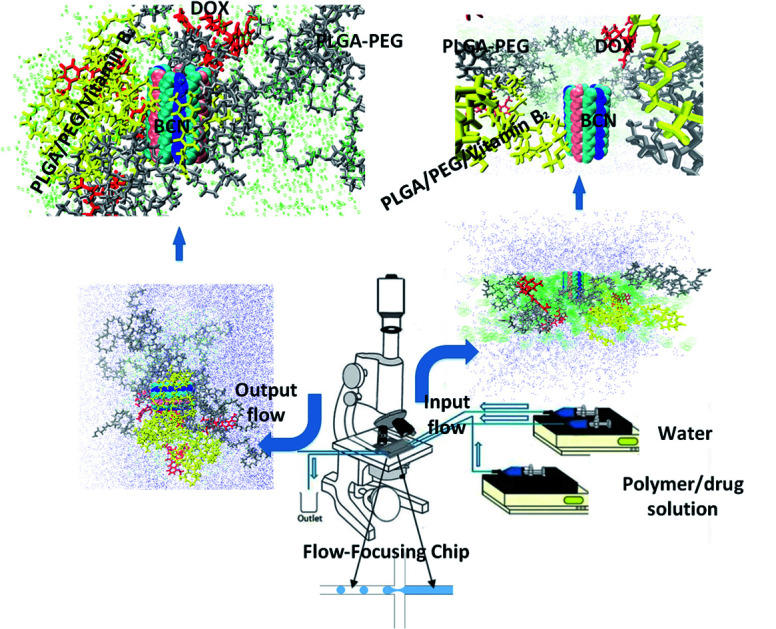

## 1 Introduction

Doxorubicin (DOX) is a chemotherapy drug for numerous cancers including breast, ovarian, bladder, and lung.^1,2^ The structure is given in Fig. S1† (ref. 1). Targeted drug-delivery can decrease unwanted damage to non-cancerous cells and make the most of the therapeutic responses of the drug.^3–8^

Synthetic and biodegradable polymers have been extensively used as a carrier for drugs, genes, and molecular imaging agents. Poly[lactic-*co*(glycolic acid)], PLGA, is reputed for its biocompatibility, nontoxicity, favorable degradation kinetics, and more importantly, it is a FDA-approved carrier. Not only hydrophobic and hydrophilic drugs, but also amphiphilic anticancer drugs can be entrapped in PLGA nanoparticles using either the nanoprecipitation method or the emulsification-solvent-diffusion methods.^9^ PEGylation of PLGA-based nanocarriers can help prolong their time in the blood circulation, and improve the drug payload, solubility, and kinetic stability while enhancing the targeting index and the accessibility of the carrier toward the tumor site.^10^

Vitamin B_2_ (riboflavin (RF)) with properties such as antioxidant and anti-inflammatory is necessary for normal immune function. RF deficiency due to unbalanced diet may result in oxidative damage, cell cycle arrest, and cell stress response, weakening iron absorption, causing hearing loss, and cranial nerve deficits.^11^ RF is a micronutrient playing roles in a variety of redox reactions through the cofactors flavin mononucleotide (FMN) and flavin adenine dinucleotide (FAD), which are essential electron carriers during cellular respiration and energy production. Moreover, the derivatives of RF have been applied as tumor targeting ligands *in vitro* and *in vivo*, which has shown the enhanced tumor-specific accumulation of riboflavin-targeted nanotubes carrying paclitaxel in MCF-7 cells12 as well as riboflavin-targeted liposomes in murine A431 and PC3 tumor xenografts.^12^ The success in the practical implementation of anticancer nanomedicine is closely related to the drug delivery system. The major objective of delivery systems is to enhance the specificity of drug delivery to target tissues, minimize the adverse effects, prolong its half-life, protect the drug's molecule, and increase its biocompatibility.^13^

Nanotechnology-based drug-delivery systems have assisted the pharmaceutical methods in smart controlling of the drug release, defensive carrying of pharmaceutical molecules. Moreover, the efforts in size control of nano-shell structures pass help the drugs to pass through the biological barriers and keep stable in the bloodstream.^14^ Carbon nanotubes (CNT) have been applied for the targeted release of several drugs such as curcumin, doxorubicin, and others. Carbon nanotubes can have both a drug carrier and a sensor role.^15–17^ There evidences that CNT can enter vertically into the membranes without damaging cell membranes *via* the endocytosis process. CNTs can encapsulate the drug and deliver them to the target.^18–20^ The large surface area of carbon nanotubes (CNT) can offer multi-attachment sites for the drugs. A drug delivery system with doxorubicin and carbon nanotubes has highlighted the effect of size; the doxorubicin (DOX) molecule can inter CNT, once the diameter is larger than 1.25 nm. However, for smaller CNT diameters the drug covers the outer surface of CNT.^21^ The surface functionalization of CNT can enhance the low dispersion of pristine CNTs in aqueous solution.^22,23^ CNT-based polymer nanocomposites have been of great interest in developing a new generation of materials exhibiting unique combinations of properties and functionalities.^24^

Khoshoei *et al.* (2020) have investigated the doxorubicin loading and delivery with carbon nanotube. According to their results on gyration radius van der Waals interaction, and RDF analyses have found that pH-responsive polymers are appropriate for DOX delivery.^8^ The intrinsic lack of control over mixing in the conventional bulk synthesis methods typically results in the production of nanoparticles suffering from wide size distributions and deteriorated physicochemical properties, which may hamper the particles to reach clinical trials. However, microfluidics has shown to be an efficient bottom-up technique for synthesizing nanoparticles with excellent control over composition, morphology, size, and size distribution.^25^

In this work, we simulated the facilitation of last-generation CNT for DOX loading in the PLGA–PEG–riboflavin nanoparticles. This is the first combination of PLGA–PEG–riboflavin with last-generation CNT, in which some portion of carbon atoms were replaced by elements such as nitrogen, bromine, and phosphorous. Because DOX is hydrophobic, PLGA is selected and PEG is aimed to increase its biocompatibility with the bloodstream and RF has been applied as tumor-targeting ligands. The compositional ratio has been taken from its optimization in our previous work.^26^ Moreover, the second novelty of this work is arisen from the case domain, which is microfluidic system. To the best of our knowledge, this is the first approach to consider a controlling phase. Microfluidics is a cutting-edge technology, which is reputed for a large surface to volume ratio and precise control over mixing and heat and mass transfer. With the microfluidic system, a narrow size distribution for the synthesis of nanoparticles would be possible. To model the self-assembly of RF-conjugated nanoparticles in the microfluidics, the interface method has been employed. Herein, the role of molecular dynamics (MD) simulation will be emphasized due to its prediction capabilities prior to experimental study, which not only saves time, material, lab work, and analysis, but also it is promising to reduce the drug side effects on patients. It should be noted that the CNT has high production costs. Moreover, MD simulation can unravel the challengeous details of the interactions between the drug and carrier at molecular level, which is difficult to be achieved through experiments alone. In this work, MD analyses are organized to investigate the hydrophilicity of the complexes, stability, and energy analysis. For the first time, the stability and size of PLGA-based nano-carriers have been engineered using doped-nanotube. The novel method for engineering of nano-carrier's properties has been investigated using the last generation of nanotubes and microfluidic method, and a new method for tuning the properties of nanocarriers has been introduced that can be developed to other nano-carriers. This study paves the way for molecular engineering of the properties of nano-carriers for better efficacy in cancer treatment. The future research works are focused on revealing other aspects of the microfluidic environment on the drug delivery systems.

## 2 Material and methods

### 2.1 Materials

The chains were sequentially optimized using Avogadro and HyperChem software's UFF and OPLS-AA force field. The molecular structure of DOX was obtained from www.rcsb.org. The initial molecular structures of CNTs were designed by Nanotube_Modeler_1.7.9.^27,28^

The effect of dopant concentration on drug stability in nanocarriers has been investigated in nan-microfluidic method. Using PMF calculations, Gibbs free energy has been performed in different concentrations of phosphorus and nitrogen and the concentration of 10% has been determined as the optimum concentration for drug loading. Our data with different contents of dopant (Table S1†) has shown that the once CNT is enriched by ten percent of dopant, the Gibbs free energy of doxorubicin loading on CNT-PLGA-PEG-RF is the least. Actually, this is the optimum concentration of dopant. For doped structures, ten percent of carbon atoms were replaced by the dopant as an example, for N-doped CNT structures, 10% of carbon atoms were replaced by nitrogen atoms.

In the next step, optimizations were carried out using Gaussian 09 software by considering the b3lyp function and 6-31^+^G* basis set. To carry out the simulations we used GROMACS 2019.5 (OPLS-AA force field) in the EM (10 kJ mol^−1^ nm^−1^ minimum force), *NVT* (500 ps), *NPT* (500 ps) and then the MD (100 ns) simulation in 2 fs time steps. All cases contained 30 600 water molecules (SPC/E water model) in boxes with 3 × 3 × 30 nm^3^.

The cutoff radius was adjusted to 1.4 nm for the van der Waals and Coulomb interactions. We used Coulomb energy algorithm and Particle Mesh Ewald (PME). The MD simulation step was carried out with isotropic Parrinello–Rahman algorithm at 1 bar and with Nose–Hoover (velocity-scaling algorithm in *NVT* and *NPT*) at 300 K. The constraint algorithm was based on the links algorithm that was only used for hydrogen bonds. The partial atomic charges of the structures are calculated using Gaussian software (pop = esp).

In our simulation systems, the total number of polymer chains is ten, in which the number of PLGA–PEG chains ratio to PLGA–PEG–RF chain numbers is defined as the PP : PPR ratio. Our previous study, on poly-lactic-*co*-glycolic acid (PLGA) hydrophobic core with poly-ethylene glycol (PEG) hydrophilic shell and varying numbers of riboflavin (RF) molecules as ligands, showed that the minimum Gibbs free energy (−9.3514 ± 0.03 kcal mol^−1^) is attributed to PP : PPR = 8 : 2. This result is related to polymer molecular weight of PLGA 3 kDa–PEG 2 kDa.^26^

In the bulk method, continuous mixing assists the diffusion of water molecules to the polymeric phase and precipitation of nanoparticles, while in the microfluidics, the interface of parallel streams limits the phenomenon and leads to smaller and more compact particles. To compare the two systems, molecular dynamic simulation has been performed to mimic the nanoprecipitation in the two following approaches:

(1) Bulk self-assembly: all the polymeric solutions have been added randomly to water molecules in the simulation boxes.

(2) Microfluidic self-assembly: an interface has been created between the phase containing polymeric chains with water phase, which controls the size of nanoparticles in flow-based synthesis. The scheme representing the flow-focusing design of the microfluidic drug delivery system for DOX has been shown in Fig. S2.†

### 2.2 Molecular dynamics

Newton's motion equations, for all system particles, were applied in Molecular dynamics that can provide a set of consecutive atomic positions to predict the upcoming moments based on the present condition.^29^ The molecular dynamic study was done in various stages. In the first stage, the initial configuration of the particles was obtained. In the second stage, calculation of neighbors' lists was made within the force range of the targeted atom in the system. Finally, according to the applied force on each atom based on the configuration, initial conditions and acceleration of each particle, calculations were done through integral methods. Newton's motion equations were solved in short time intervals (1–10 fs) for an integrated calculation. In each step, calculation of the applied force on atoms with the present situation and velocities was done to reach the upcoming positions and velocities in the following step. A set of forces was considered as the atoms displace to new positions and the process will be repeated. In this respect, molecular dynamics simulation can be a beneficial path to describe the dynamic variables changes by passing time.^30,31^

#### 2.2.1 Force fields

The accuracy and validity of simulated results were determined by using force fields. Interactive energies (potential energy) of inter-particle will be changed by changing their distances. Eqn (1) presents the potential function; while the force function of each “*i*” atom in a N-atom system is achievable from eqn (2), which is extracted from the potential function. These equations were concomitantly solved. In addition, the force can be allocated to time and the atomic position by using eqn (3).^32,33^ Simple potentials such as the hard sphere potential can be applied in the primary molecular simulation. In this model, it was assumed that the particles move in straight lines with a constant velocity. When the distance of spheres becomes equal to the sum of their radii, relatively elastic collisions will happen. Then, a new velocity will be calculated based on the principle of conservation of linear motion size. By using the hard-sphere model, beneficial results will be achievable, although in atomic or molecular system simulations it is not ideal.

Based on the van der Waals potential, as interatomic or intermolecular distances vary, their forces change. However, no force was considered among particles unless they collide with each other. van der Waals potential was represented in eqn (4), where “*σ*” shows the potential good depth and “*q*” denotes the distance at which the potential becomes zero. Calculations can be used to specify these parameters by fitting with laboratory data or exact quantum chemistry. “*r*” illustrates the distance between two atoms, and their inter-atomic potential was shown by “*V*”:^34^

1 *U* = *u*(*r*)

2
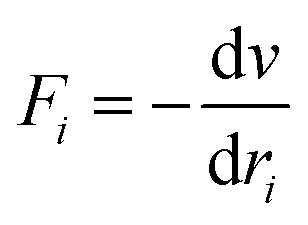
3
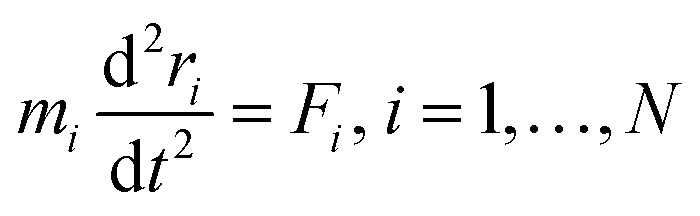
4
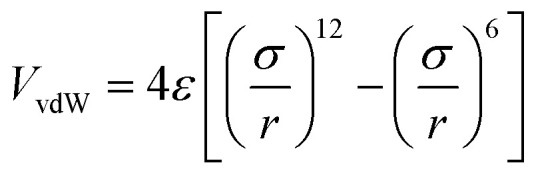


Drug diffusion coefficient can be calculated using eqn (5) and (6). Mean-square displacement (MSD) was calculated to reveal the drug diffusion coefficient; coordinates of atoms are also in view as “*r*” while “*t*” shows time. After MSD calculation, diffusion coefficient can be calculated for a three-dimensional system by using Einstein's relation (eqn (6)).^35^5
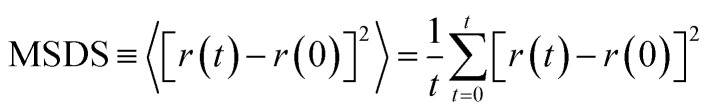
6
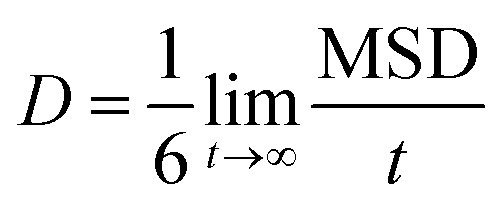


#### 2.2.2 System preparation

According to similar structures available in the OPLSaa force field, charge and other relevant parameters of the nanostructured functional groups were defined. Calculation of non-bonding interactions (*i.e.*, electrostatic and van der Waals) was done by applying Lenard-Jones and Columbian potential models. The input structure was prepared by implementing the OPLSaa force field. All molecules were placed in the box and the tip3p water model was utilized as a solvent to obtain molecules parameters (changed to script format). 50 000 steps were employed to minimize the energy of all simulation systems. To omit van der Waals interactions and create hydrogen bonds between water molecules and other species, the steepest descent method was applied. Nose–Hoover algorithm was used to increase the temperature of the system from 0 to 310 K in 100 ps in a constant volume in the next stage. Moreover, temperature-coupling systems were considered as 0.5 ps. Then at a constant pressure in 200 ps, the system was balanced. The Parrinello–Rahman algorithm was employed to balance the system pressure. The temperature of 37 °C for 50 ns was used in the MD simulation. The cut-off distance was set to 1.2 and the electrostatic force calculation was conducted by employing Particle Mesh Ewald (PME). The LINCS (linear constraint solver) algorithm was adopted for maintaining the bond lengths; while the bonds engaged in hydrogen atom were limited by applying the SHAKE algorithm, which will accelerate the calculations.

For Gibbs free energy, the umbrella sampling technique has adopted. Umbrella simulation input structures are the samples that are resulted from the output of the MD simulation. First, pull code was used to separate one of the 10 polymers from aggregation. Second, 100 configurations were extracted from pull code simulation.

After applying pull code for polymer strand, it restrained at increasing center-of-mass (COM) distance from polymer strands that leads to the generation of various configurations for each location. The PMF curve can be extracted in the restrain stage using the polymers strands' positions to the COM. In another word, integration of PMF corresponding to the series of configurations. Finally, Gibbs free energy was obtained by WHAM analysis^36,37^ on all configurations. The WHAM analysis method is a very powerful technique based on the estimation of the statistical uncertainty of the probability distribution provided by the umbrella method.^38,39^

## 3 Results and discussion

### 3.1 Snapshots of polymer/drug solutions

**Fig. 1:**
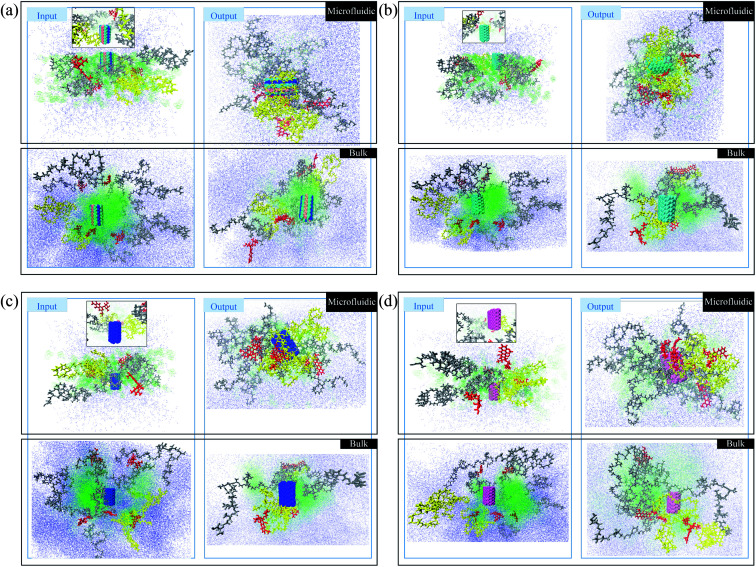
(a) Snapshots of PLGA/PEG/RF for dual delivery of BCN-doped CNT and DOX in bulk and microfluidic systems. (b) Snapshots of PLGA/PEG/RF for dual delivery of CNT and DOX in bulk and microfluidic systems. (c) Snapshots of PLGA/PEG/RF for dual delivery of N-doped CNT and DOX in bulk and microfluidic systems. (d) Snapshots of PLGA/PEG/RF for dual delivery of P-doped CNT and DOX in bulk and microfluidic systems.

**Fig. 2:**
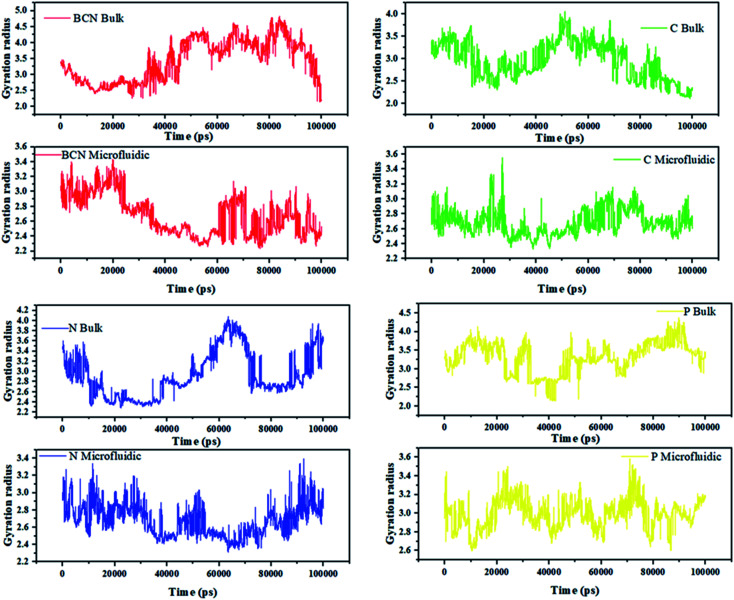
The gyration radius trends of the DOX/CNT-based structures/PLGA/PEG/RF in bulk and microfluidic systems.

In this section, a preliminary test is performed to compare the photos of polymer/drug solutions in bulk and microfluidic systems. The red molecules represent doxorubicin (DOX) molecules. The yellow polymer chains are PLGA–PEG–Vitamin B_2_, and the gray polymers are PLGA–PEG. Water and acetonitrile molecules are blue and green, respectively. In Fig. 2a, boron carbon nitride (BCN) is also present in blue/green/pink color. According to Fig. 1a, the polymer/drug molecules in the output surround the BCN and it is notable that the aggregation is stronger in the microfluidic system. Moreover, water molecules distribution is not uniform in the input of the process of the bulk system, which is not seen in the microfluidic system with better control of the fluid flow.

In Fig. 1b, in addition to polymers and DOX, the carbon nanotube is also present in green color. According to Fig. 1b, the polymer/drug molecules in the output surround the carbon nanotube and it is notable that the aggregation is stronger in the microfluidic system. Moreover, the water molecules distribution in the input of the process of the microfluidic system is more uniform than the corresponding ones in the Fig. 1a.

In Fig. 1c, in addition to polymers and DOX, the nitrogen-doped carbon nanotube is also present in blue color. According to Fig. 1c, the polymer/drug molecules in the output surround the carbon nanotube and it is notable that the aggregation is stronger in the microfluidic system. Moreover, the water molecules in the input of the process of the bulk system are more densely surround other molecules in comparison with its equivalent ones.

In Fig. 1d, in addition to polymers and DOX, the phosphorous-doped carbon nanotube is also present in pink color. According to Fig. 1d, the polymer/drug molecules in the output surround the carbon nanotube and it is notable that the aggregation is stronger in the microfluidic system. Furthermore, the water molecules distribution in the input of the process of the microfluidic system is more homogeneous than the corresponding ones in the Fig. 1d. In total, the microfluidic system provides a stronger aggregation that is because of the astonishing merits of microfluidics, which can be seen in the reduction of the mixing time, and accordingly the nucleation is executed in submicron sizes. With finer nucleons, the stability of the system is the higher. Furthermore, solvent flow in the microchannel creates shear stress forces and shearing forces causes the molecules of weak clusters to be separated from each other and only stable clusters can be remain. Therefore, in addition to mixing time, shear stress also tunes the stability and particle size and improves nanoparticle's properties.

### 3.2 Hydrophilicity of PLGA–PEG/CNT-base structures

It has been reported that the hydrogen bond between the drug and the carrier can highly affect the drug-loading rate.^8,40^ In hydrogen bonding, the angle between receptor–acceptor pair is less than 30°. Moreover, the higher the number of hydrogen bonds, the higher the hydrophilicity of the drug delivery system, results in better solubility of the drug. In Fig. S3,† the average number of hydrogen bonding is given for eight samples with the presence of pristine and doped CNT in bulk and microfluidic systems. According to Fig. S3,† the microfluidic system increased the hydrophilicity of micelles in the presence of BCN, and phosphorus doped CNT samples with regard to their bulk systems. To have a more precise analysis of the hydrogen bonding between the DOX and CNT-based nanostructures, another graph has been provided. According to Fig. S3,† BCN–DOX contribution is the largest among all other nanotube–DOX interactions. It is notified that the nanotube–DOX interaction magnitude is very low compared to the hydrogen bonding average number in the micelle–drug pairs.

### 3.3 Stability

To determine the stability of samples, the gyration radius, root-mean-square deviation (RMSD), and Gibbs free energy analyses have been investigated.

#### 3.3.1 Gyration analysis

The *R*_g_ indicates the accumulation of polymer particles around its center of gravity at different times. The lower the *R*_g_, the greater the accumulation of particles around the center of gravity.^41–43^ In case the (*r*_*i*_ − *r*_cm_) communicates the difference between the particle *i*, and the particle center of mass at that point and the *R*_g_ from the connection 1 is calculated. The *R*_g_ of the complex can be calculated from eqn (7):

7 *R*_g_ = (1/*N*_*i*_∑(*r*_*i*_ − *r*_cm_)^2^)^1/2^

In the gyration analysis as the first estimation, the clue is that the less the gyration radius, the more stable the complex. The gyration radius-analysis data for the whole samples has been summarized in Table 1. Accordingly, the sample with BCN is the most stable complex in both microfluidic and bulk systems. The results show that for the microfluidic system, three out of four samples face the reduction in gyration radius, which is more promising in comparison with the results of the bulk system. Moreover, this analysis suggests for microfluidic samples, the significance of sample stability is as follows: BCN > CNT > N-doped CNT sample and for the bulk system, the most stable complex is BCN and the after, only the CNT sample is stable. In the meantime, although the CNT and N-doped CNT samples in the bulk system are more hydrophilic than the sample containing BCN, the latter sample is a step nearer to the optimum point.

**Table 1:** The difference between initial and final gyration radius and the final gyration radius of the complex resulted from the conjugation of DOX/CNT-based structures with PLGA/PEG/RF

Analysis	Gyration			
System	Microfluidic		Bulk	
Samples	R0–R100 (nm)	R100 (nm)	R0–R100 (nm)	R100 (nm)
BCN	0.53772	2.53569	1.17895	2.22511
C	0.27319	2.60931	0.91054	2.33181
N	0.18895	2.81491	−0.24661	3.68484
P	−0.20297	3.16361	−0.18114	3.45944

Fig. 2 can indicate the gyration radius trends of the DOX/CNT-based structures/PLGA/PEG/RF in microfluidic and bulk systems up to 100 ns. According to Fig. 2, the gyration radius trends of the whole samples face more changes in bulk systems compared to microfluidic systems, which highlight the better control of mixing and self-assembly of the polymeric composites. The reduction in gyration for BCN sample in both microfluidic and bulk system is observable. For bulk BCN sample, in total, the trend in gyration radius is descending, but there is a region in time between 40–80 ns when the increase in the gyration radius is observed. It can be seen that for CNT samples as BCN samples both bulk and microfluidic systems are stable and the gyration radius is decreased. For the CNT bulk samples, the ascending region is between 30–50 ns. For the N-doped sample, the microfluidic system is stable, while the reverse is true for the bulk system and not only there are two regions of instability in the bulk system (40–65 ns and 80–100 ns), but also there is a region of instability in microfluidic system (80–100 ns). For the P-doped CNT samples, neither the microfluidic systems nor the bulk systems are stable. There are three main regions of instability (0–10 ns, 40–60 ns, and 70–90 ns) in the bulk system. The instability regions of the bulk P-doped system are 10–25 ns, 40–50 ns, and 60–70 ns.

Song Yang Khor *et al.* (2018) show that during self-assembly, the impact of flow conditions strongly depends not only on the particle size, but also on the particle surface chemistry. Their results are in agreement with the gyration radius analysis results.^44^

#### 3.3.2 Root-mean-square deviation (RMSD) analysis

**Fig. 3:**
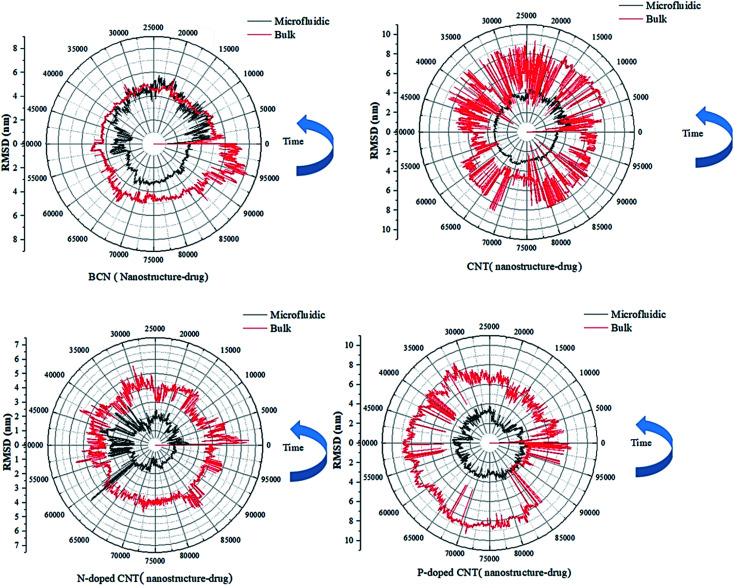
The RMSD curves of the DOX–nanotube interaction of DOX/CNT-based structures/PLGA/PEG/RF in bulk and microfluidic systems over 100 ns.

The second criterion of stability in molecular dynamics is the root-mean-square deviation (RMSD) analysis, which represents the deviation of particle position relative to their reference position at each time. The narrower RMSD range, the more stable the sample. However, the RMSD results are confirmed by gyration radius analysis and should be favorable in regard to hydrophilicity. Fig. 3 shows that during the 100 ns, RMSD values DOX–nanotube in the bulk systems exceed the RMSD value of the microfluidic system. In approximately 2.5% of the times, for systems containing N-doped, there is a higher RMSD value for the bulk system compared to the microfluidic system. For the samples containing N-doped CNT in the microfluidics system, from 35 ns to 65 ns, the RMSD values face very changes. This result can show that for systems with these functional groups, due to their interactions, a morphological change can be taken place. The larger mean hydrogen bonding of this structure, which is observed in Fig. S3,† confirms this phenomenon. Moreover, for BCN-doped and CNT samples in the microfluidic system, the RMSD values are larger in the first 50 ns compared to the second half of the time. As an instance, BCN-doped in the microfluidic system, RMSD values are lower than 6 nm in the first half of the time, and they lower than 4 nm in the second half. The lowest RMSD values of the DOX–nanotube during 100 ns is observed in the microfluidic system with the presence of P-doped CNT, which are below 4 nm. For bare CNT, in the first 30 ns (30% of the whole time), the RMSD values are 4 nm, and in the 70% of the times it is below 4 nm.

According to Table 2, the particles in microfluidic systems have fewer movements compared to bulk systems, which is due to the laminar flow that is dominant in microfluidic systems. For micelle–drug, the microfluidic BCN-doped CNT–DOX is the most stable complex, which is in complete agreement with the gyration radius analysis and its average number of hydrogen bonds is satisfactory. The microfluidic P-doped CNT–drug has a narrow RMSD range, but the gyration analysis rejects its stability. The RMSD stability of microfluidic CNT and N-doped CNT are also confirmed by the gyration analysis and they have favorable hydrophilicity.

**Table 2:** The minimum and maximum values for RMSD for micelle–DOX and DOX–nanotube of the DOX/CNT-based structures/PLGA/PEG/RF complex in bulk and microfluidic systems

Analysis	RMSD			
System	Microfluidic		Bulk	
Samples	Min	Max	Min	Max
BCN (micelle–drug)	0.0001845	4.8306155	0.0000247	6.3340025
BCN (nanostructure–drug)	0.000161	5.7536497	0.0000566	8.2631464
CNT (micelle–drug)	0.0000004	5.1942415	0.0002146	6.8699155
CNT (nanostructure–drug)	0.0000006	4.9909511	0.0001584	9.4036846
N-Doped CNT (micelle–drug)	0.0001331	5.8436046	0.0000414	6.0707288
N-Doped CNT (nanostructure–drug)	0.0000081	5.9250174	0.0000418	6.556612
P-Doped CNT (micelle–drug)	0.0000486	5.0908384	0.0001697	6.6743522
P-Doped CNT (nanostructure–drug)	0.0000469	4.1120777	0.0002979	9.6248369

In RMSD test, it is possible to analysis the stability of the nanostructure–drug attachment. The microfluidic system has the following order: P-doped CNT > CNT > BCN > N-doped CNT; the reverse order of stability is reported for bulk system.

#### 3.3.3 Gibbs free energy analysis

As it is well known, the best criterion for determining the stability of any given complex is Gibbs free energy. Herein, the Gibbs free energy calculations using the umbrella sampling technique has been performed and the results are presented in Table 3. Accordingly, for each complex, the microfluidic system provides an environment toward higher stability. Moreover, the presence of dopants has significant impacts on sample stability. The Gibbs free energy confirms the findings of the gyration radius and RMSD, which introduce the microfluidic system containing BCN, is the most stable one. In the system that CNT is doped with nitrogen, the complex is the least stable among the CNT and doped-CNT samples. The effect of flow on the stability of nanoparticles in seen in the work of Mai N. Vu *et al.* 2020 who have investigated the effect of flow condition on the self-assembly and drug encapsulation. They asserted that the shear force of blood flow may be strong enough to detach a proportion of nanoparticles possessing weak cellular interactions with neutrophils and monocytes.^45^

**Table 3:** Gibbs free energy for samples containing (doped) carbon nanotube in microfluidic and bulk systems

Analysis	Gibbs free energy (k cal mol^−1^)	
(Doped) nanotube	System	
	Microfluidic	Bulk
BCN	−34.20	−22.95
CNT	−30.84	−14.62
N-Doped-CNT	−19.39	−12.64
P-Doped-CNT	−25.41	−13.46

### 3.4 Energy analysis

The binding energy between micelle and drug for four samples (BCN, CNT, N-doped CNT, P-doped CNT) was investigated. The MM/PBSA^46,47^ software was utilized to calculate the energy contribution of electrostatic and van der Waals bonds.

The data of van der Waals, electrostatic, and total energies of samples in microfluidic and bulk systems have been given in Table 4. The negative average electrostatic energy is an indication of strong attachment according to Table 4, the electrostatic energy of each sample in the microfluidic system is larger than the equivalent ones in the bulk system. Therefore, DOX in microfluidic systems can be more strongly adhered to the micelles. The highest electrostatic interaction can be seen between polymeric micelle and DOX in the presence of BCN. This result is true for gyration radius analysis and RMSD. Moreover, the H-bonding test has shown the most hydrophilic sample is BCM in the microfluidic system. The order of significance of electrostatic energy between micelle–DOX microfluidic system containing nanostructures is as follows: BCN-doped CNT > P-doped > CNT > CNT > N-doped CNT.

**Table 4:** The energy interactions between micelle–DOX of the DOX/CNT-based structures/PLGA/PEG/RF complex in bulk and microfluidic systems

Scale	Micelle	van der Waals energy			Electrostatic energy			Total energy		
		Max	Min	Avr	Max	Min	Avr	Max	Min	Avr
Micro	BCN	−76.354	−799.27	−478.289	18.277	−717.169	−363.872	−71.689	−1425.529	−842.160
	C	−46.852	−756.181	−321.035	20.998	−599.966	−259.822	−67.07	−1296.078	−580.857
	N	−59.393	−589.682	−337.408	41.236	−424.338	−226.866	−43.087	−913.451	−564.274
	P	−56.685	−666.608	−377.717	28.564	−556.896	−333.501	−48.262	−1194.34	−711.218
Bulk	BCN	−4.68	−788.365	−369.611	21.084	−608.279	−259.258	−8.115	−1357.059	−628.869
	C	−5.735	−728.538	−401.517	21.639	−613.747	−270.094	−10.965	−1253.43	−671.612
	N	−39.179	−878.533	−554.499	1.538	−507.19	−281.258	−70.464	−1299.294	−835.757
	P	−25.393	−661.968	−252.147	16.363	−420.062	−146.849	−35.199	−958.316	−398.996

The order of significance of van der Waals interactions between micelle–DOX in the microfluidic system is the same as the electrostatic interaction. For the complex containing BCN-doped in the microfluidic system, the largest mean electrostatic (−363.872 kJ mol^−1^), van der Waals (−478.289 kJ mol^−1^), and total energy (−842.160 kJ mol^−1^) between micelle–DOX have be reported. The minimum mean electrostatic (−259.258 kJ mol^−1^), van der Waals (−369.611 kJ mol^−1^), and total energy (−628.869 kJ mol^−1^) between micelle–DOX have been reported.

Table 5 shows that for nanotube–drug interactions, the average electrostatic energy for the microfluidic samples is stronger than the bulk samples. However, there is an exception for the BCN sample that in the bulk system, the electrostatic energy is higher than the microfluidic sample. The π–π interactions in the complexes with aromaticity contribute to a great portion of the van der Waals interactions and highly affects the DOX loading process.^48^ In the complexes containing BCN-doped CNT, N-doped CNT, CNT, van der Waals interaction dominates the DOX–nanotube interaction, while for P-doped CNT both the electrostatic and van der Waals are important. Phosphorus-doped carbon nanotube in the microfluidic system has the largest electrostatic interaction (−186.835 kJ mol^−1^) with the DOX, while in the bulk system, it has the minimum interaction (−0.016 kJ mol^−1^) with DOX. This finding highlights the capabilities of the microfluidic system with P-doped CNT.

**Table 5:** The energy interactions between DOX–nanotube of the DOX/CNT-based structures/PLGA/PEG/RF complex in bulk and microfluidic systems

Scale	Nano tube	van der Waals energy			Electrostatic energy			Total energy		
		Max	Min	Avr	Max	Min	Avr	Max	Min	Avr
Micro	BCN	−25.459	−144.529	−102.424	3.907	−9.681	−2.450	−25.108	−149.356	−104.874
	C	−8.567	−356.982	−217.614	28.331	−29.401	−4.788	−6.242	−373.564	−222.402
	N	−0.004	−228.353	−147.293	0	−6	−2.987	−0.004	−228.353	−147.293
	P	−20.603	−297.198	−226.863	−43.205	−284.104	−186.835	−82.335	−536.118	−413.698
Bulk	BCN	−0.008	−283.974	−206.475	7.353	−18.05	−6.418	0.243	−293.96	−212.893
	C	−0.003	−313.396	−107.705718	0	−2	−1.004	−0.003	−313.396	−107.705
	N	−0.238	−171.344	−127.705	32.76	−22.073	−0.693	0.451	−185.605	−128.399
	P	−0.002	−49.068	−1.825	27.215	−34.144	−0.016	8.929	−45.364	−1.842

In addition, the aromatic–aromatic interactions between the drug and nanotube can be determined by van der Waals energy, which is the highest (−226.863 kJ mol^−1^) in the complex for dual delivery of P-doped CNT–DOX in the microfluidic systems. Interestingly, this complex in the bulk system has a minimum van der Waals energy of −1.825 kJ mol^−1^.

### 3.5 Radial distribution function (RDF)

RDF is the probability of finding a molecule at a spherical shell of a certain thickness at a distance (*r*) from the surface of the nanotubes. The position of the first peak in the plot of *g*(*r*) *versus r*, characterizes the closest distance between the two groups. In the meantime, the probability that one group appears at this distance would be demonstrated by the first peak height in this plot.^49^

**Fig. 4:**
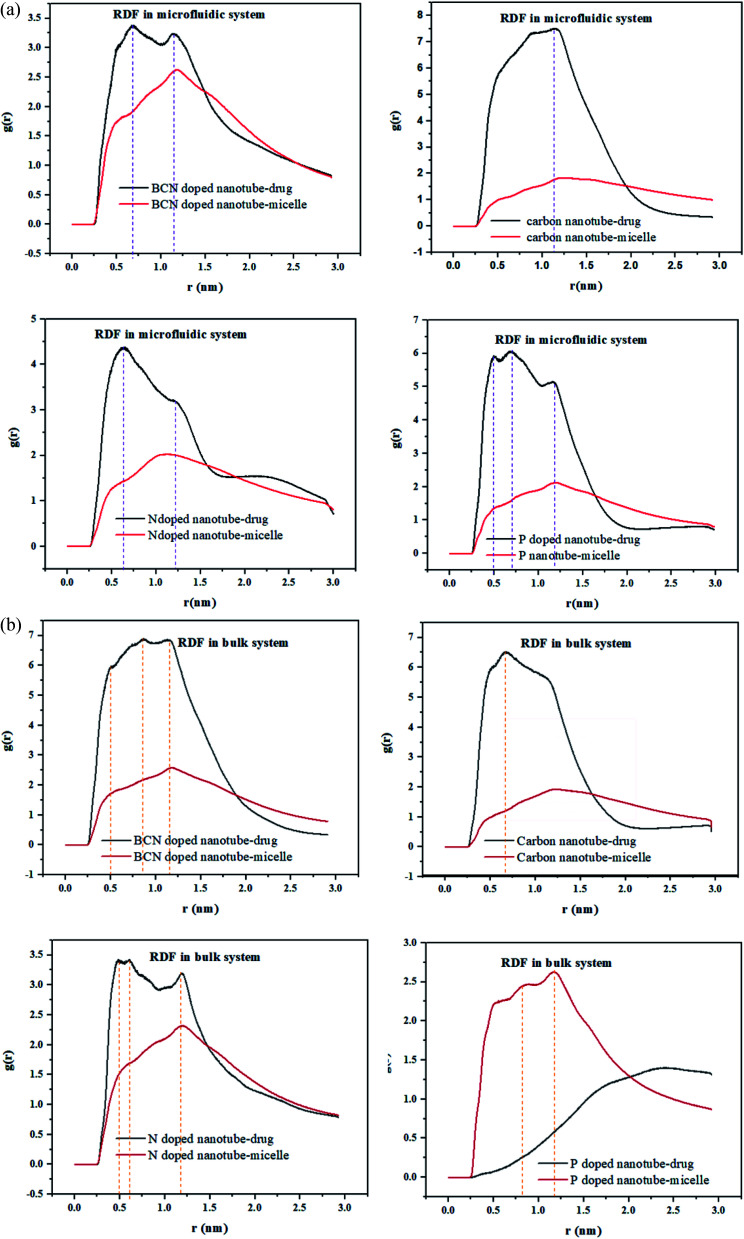
(a) The radial distribution function of the DOX/CNT-based structures/PLGA/PEG/RF in the microfluidic systems. (b) The radial distribution function of the DOX/CNT-based structures/PLGA/PEG/RF in the bulk systems.

The radial distribution function (RDF), *g*(*r*), of the drug and the polymeric micelle molecules with respect to the surface of the nanotubes is calculated using gmx_rdf analysis. The first peak positions of the nanotube–drug are shown in Fig. 4a. DOX is observed at more than 1 nm apart from the CNT, while for the doped CNT, the DOX is closer to the nanotube, which is due to the functional groups incorporated in CNT.

It is interesting to acknowledge that in the DOX delivery system of PLGA–PEG–riboflavin/CNT based structures, the stronger attachment of CNT is associated for the polymeric micelle, or to the drug. For all samples in the microfluidic system, the higher nanotube–drug affinity compared to nanotube–micelle is indicated by sharper peaks in Fig. 4. This result magnifies the positive effect of the nanotubes in addition to the DOX delivery system. Among them, the BCN sample has a balanced affinity to both micelle and drug. This finding is confirmed by the gyration radius and RMSD analyses, which validate that the BCN sample has the most stable micelle–drug and favorable drug –nanotube stability.

In the bulk system, all samples except P-doped CNT exhibit a higher affinity toward the drug compared to micelle. Fig. 4b can show that the P-doped CNT, which is the most instable according to the gyration radius and RMSD analyses, does not have an affinity to the drug in the bulk sample.

### 3.6 Solvent accessible surface area (SASA)

A nano-scale insightful measurement, which shows the conformation change and self-assembly is SASA. The mean SASA value can be obtained by the following equation:

8 Contact area (*t*) = 1/2(*S*(0) − *S*(*t*))

where *S*(0) is representative of the SASA value at the beginning of the simulation and *S*(*t*) indicates the SASA value at any given time.

Herein, the copolymers are amphiphilic and form nano-aggregations immediately in contact with water molecules. Therefore, the total trend of solvent-accessible surface area over 100 ns (Fig. S4† and 5) is descending. Moreover, the fluctuations can be attributed to the conformational changes of the strands. Fig. S4† shows that the SASA curve of DOX in the presence of different (doped) CNT is varied from 25.373 nm^2^ (in the presence of N-doped CNT in microfluidic system) to 40.005 nm^2^ (in the presence of P-doped CNT in the bulk system). According to Fig. S4,† as an instance for N-doped CNT in the first 20 ns, a sharp decrease in SASA is observed, which is followed by another decrease around 70 ns.

**Fig. 5:**
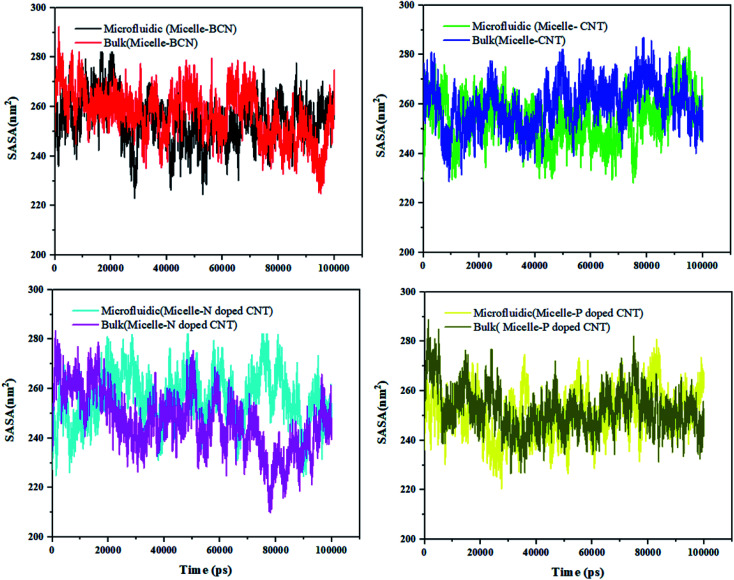
The SASA trends for micelle in the presence of doped CNT in bulk and microfluidic system.

Fig. 5 shows that the SASA curve of the polymeric micelle in the presence of different (doped) CNT ranges from 209.914 nm^2^ (in the presence of N-doped CNT in the bulk system) to 293.912 nm^2^ (in the presence of BCN in the microfluidic system). For polymeric micelle, the SASA magnitudes and fluctuations are larger than the SASA trends of DOX, which is confirmed by the larger number of H-bonds in Fig. S3.†

To gain knowledge of the average SASA over 100 nm, Table 6 is provided. In the sample with N-doped CNT in the microfluidic system, the DOX SASA mean value is minimum among the different nanotubes. While, the highest SASA mean value is associated to CNT in the bulk system. For polymeric micelle, the sample with P-doped CNT in the microfluidic system has the minimum SASA mean value and the highest SASA mean value is associated to CNT in the bulk system. In the presence of BCN in the microfluidic system, the highest SASA result has been achieved 293.912 nm^2^, which provides the highest surface area for loading the DOX.

**Table 6:** SASA analysis for DOX and micelle in the presence of doped CNT in bulk and microfluidic system

Analysis	SASA (nm^2^)					
System	Microfluidic			Bulk		
Samples	Min	Mean	Max	Min	Mean	Max
DOX (BCN)	31.424	34.56257804	39.297	31.601	34.94366953	39.071
DOX (CNT)	28.421	31.2809704	35.474	34.14	37.42637106	39.982
DOX (N-doped CNT)	25.373	28.27883032	38.977	29.211	34.18855434	39.243
DOX (P-doped-DOX)	32.167	34.70058764	39.003	28.866	33.58455434	40.005
Micelle (BCN)	222.973	254.1575241	293.912	224.847	256.859672	292.246
Micelle (CNT)	228.266	253.7237344	283.149	228.822	259.4924445	286.847
Micelle (N-doped CNT)	224.499	255.7770494	289.336	209.914	246.5965576	283.391
Micelle (P-doped CNT)	220.523	251.1034112	280.825	226.554	252.2710421	288.709

## 4 Conclusion

In this work, the effect of the microfluidic system and the addition of CNT and doped-CNT materials on the delivery of DOX have been studied. The H-bond analysis has shown that hydrogen bonding between DOX–BCN is the largest. The gyration analysis suggests for microfluidic samples, the significance of sample stability is as follows: BCN > CNT > N-doped CNT sample, and for the bulk system, BCN and pristine CNT samples are stable. The RMSD and Gibbs free energy results of the micelle–drug in the microfluidic BCN-doped CNT–DOX validate that this is the most stable complex. The lowest RMSD values (below 4 nm) of the DOX–nanotube during 100 ns is observed in the microfluidic system with the presence of P-doped CNT. All of the three stability analyses ensure that microfluidic is superior over the bulk system. Energy analysis shows that the attractions between molecules in the microfluidic systems are stronger than the equivalent ones in the bulk system. The order of significance of electrostatic energy between micelle–DOX microfluidic system containing nanostructures is as follows: BCN-doped CNT > P-doped > CNT > CNT > N-doped CNT. DOX is observed at more than 1 nm apart from the CNT, while for the doped CNT, the DOX is closer to the nanotube, which is due to the functional groups incorporated in CNT. The highest SASA is achieved for the system containing BCN (293.912 nm^2^), which provides the highest surface area for loading the DOX. The descending trends in SASA depict the self-assembly of DOX/CNT-based structures/PLGA/PEG/RF nanoparticles.

## Conflicts of interest

There are no conflicts to declare.

## Supplementary Material

RA-010-D0RA07500K-s001
