# Supplementary material for: Molecular engineering of the last-generation CNTs in smart cancer therapy by grafting PEG–PLGA–riboflavin
Source: RSC Adv. 2020 Nov 9;10(67):40637–48. doi: 10.1039/d0ra07500k (PMC9057702; doi:10.1039/d0ra07500k)
Supplement: RA-010-D0RA07500K-s001 [file RA-010-D0RA07500K-s001.pdf]

Electronic Supplementary Material (ESI) for RSC Advances.
This journal is © The Royal Society of Chemistry 2020

Supplementary information

Molecular Engineering of the Last-Generation CNTs in Smart Cancer therapy by Grafting PEG–PLGA- Riboflavin

Somayeh Sohrabi[a], Mohammad Khedri[a], Reza Maleki[b], Mostafa Moraveji[a,*]

[a] Department of Chemical Engineering, Amirkabir University of Technology (Tehran Polytechnic), 424 Hafez Avenue, Tehran, 1591634311, Iran

[b] Computational Biology And Chemistry Group (CBCG), Universal Scientific Education and Research Network (USERN), Tehran, Iran

*Corresponding author's email: moraveji@aut.ac.ir

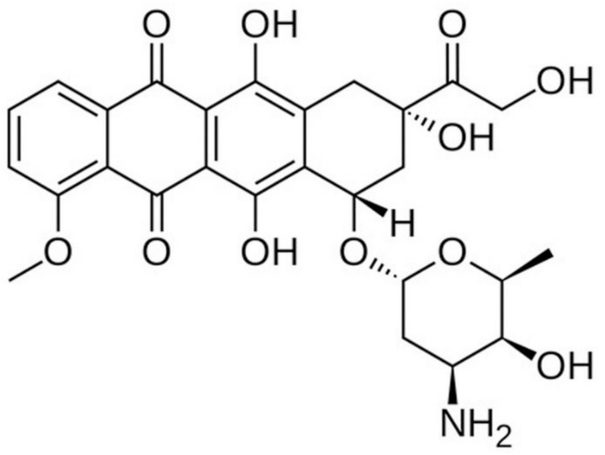

Supplementary Figure S1. Chemical structure of doxorubicin (Dox) anticancer drug [1].

Supplementary Table S1.

Dopant Concentration	Gibss Free Energy	
	N- doped-CNT	P- doped-CNT
5	-7.62	-9.04
8	-10.36	-12.14
10	-12.64	-13.46
15	-11.29	-11.91
20	-6.57	-7.89

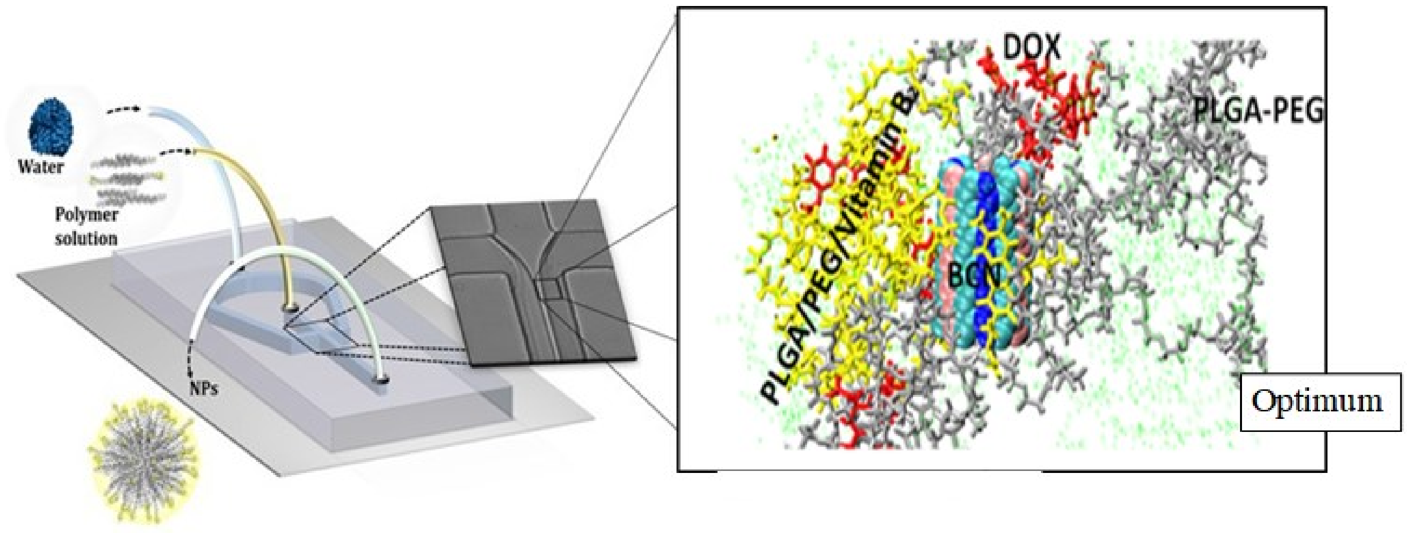

Supplementary Figure S2. The flow-focusing microfluidics system for DOX delivery

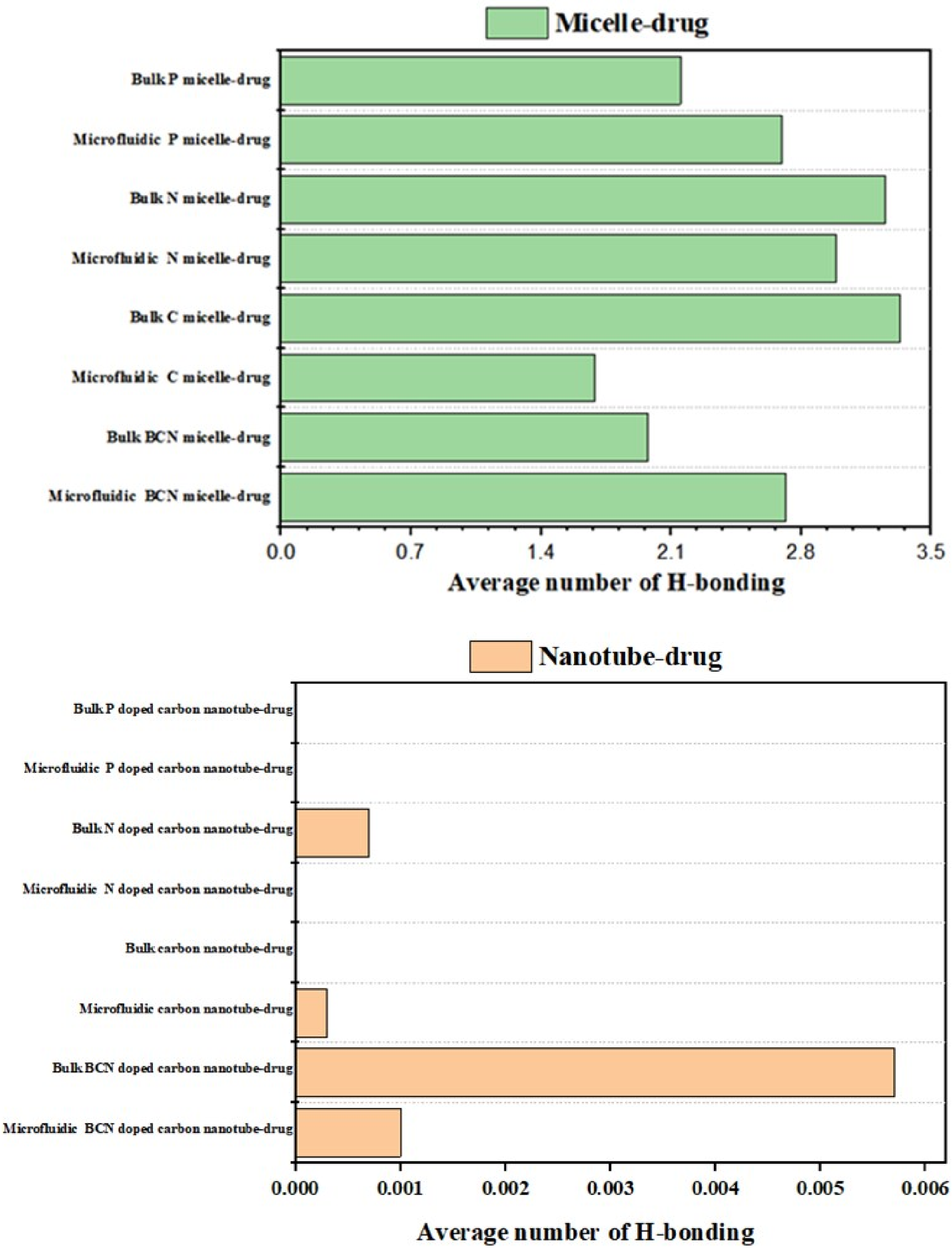

Supplementary Figure S3. Average number of hydrogen bonds in PLGA/PEG/RF@ (doped)/DOX in bulk and microfluidic systems

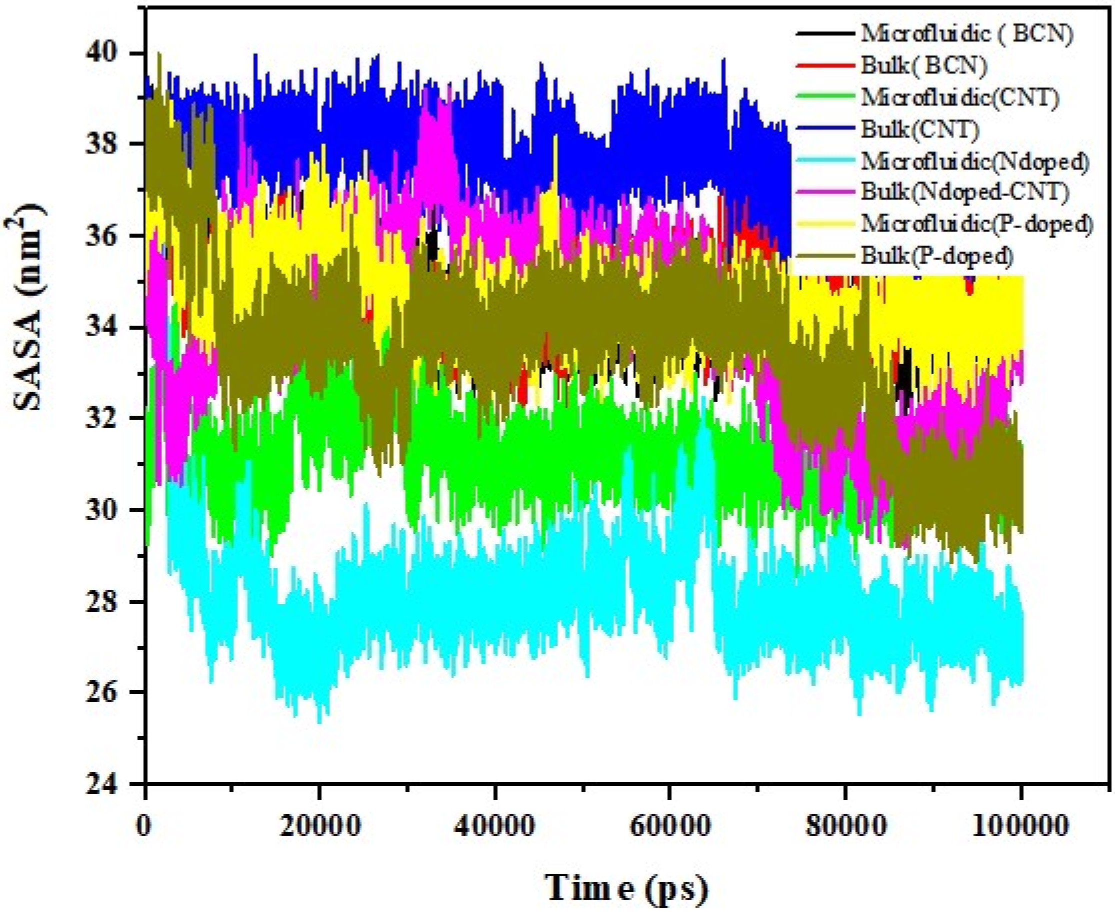

Supplementary Figure S4. The SASA trends for DOX in the presence of doped CNT in bulk and microfluidic system
